# Rural poverty and labour force participation: Evidence from Indonesia’s Village fund program

**DOI:** 10.1371/journal.pone.0283041

**Published:** 2023-06-21

**Authors:** Anu Rammohan, Achmad Tohari

**Affiliations:** 1 Department of Economics & Australia Indonesia Centre, University of Western Australia, Perth, Australia; 2 Faculty of Economics and Commerce, University of Airlangga, Surabaya, Indonesia; University of Botswana, BOTSWANA

## Abstract

Rural residents account for the bulk of poverty in developing countries. This paper evaluates the impact of Indonesia’s *Dana Desa* program (Village Fund Program or VFP) on rural poverty and female labour force participation. The VFP, introduced in 2014, was an ambitious national-level village governance program which transferred administrative responsibility and financial resources to Indonesia’s 79,000 plus rural villages, providing them with the autonomy to invest in rural infrastructure, human capital, and job creation programs. Using nationally representative data from before and after the program, we show that the implementation of the VFP was associated with an improvement in rural household’s consumption expenditure among households, particularly among agricultural households. Female labour force participation in rural areas increased by about 10 percentage points and there is also evidence of a sectoral shift away from agricultural employment towards jobs in the service sector. This improvement in labour force participation is associated with poverty reduction among rural households.

## 1. Introduction

Although significant global progress has been made over the last three decades in poverty reduction, extreme poverty continues to be widely prevalent and is predominantly found in rural areas of developing countries [1,2]. According to UN estimates, approximately 79% of the world’s poor live in rural areas, and in 2018, rural residents accounted for four out of every five individuals living below the international poverty line [3,4]. Rural poverty is also strongly associated with agricultural employment. In particular, income from farm-related activities continue to be an important source of income for agricultural households in the lowest income quintile compared to agricultural households from the top income quintile [5]. Efforts to alleviate extreme poverty in rural areas is made even more challenging due to the casual and informal nature of rural employment, and the fact that agricultural activities tend to be primarily subsistence oriented. There is also a strong nexus between agriculture, rural development and poverty. While many of the extreme poor have some access to input and output markets, a large share of their agricultural production is consumed by the household [6]. To address some of these issues, several countries have implemented targeted social welfare programs to address rural poverty and improve rural employment opportunities [6–9].

Previous research has highlighted the important role of the rural non-farm economy as a potential pathway out of poverty, and the role that policy makers can play to enhance rural economies [10]. In this paper, we evaluate one such a program- Indonesia’s *Dana Desa* program (Village Fund Program or VFP) and its role in reducing rural-urban poverty. The VFP is among the world’s largest national level rural development programs. We provide national-level evidence that the implementation of the VFP is associated with a decline in rural poverty. We then investigate the potential mechanisms that may be associated with a narrowing of rural-urban poverty. We investigate if there has been a decline in labour force participation in rural areas, particularly among females. We show that this improvement in labour force participation is associated with poverty reduction and welfare improvement among rural households.

The VFP, introduced by the Government of Indonesia (GoI) in 2014, was an ambitious national-level village governance program which transferred administrative responsibility and financial resources to Indonesia’s 79,000 plus rural villages, providing them with the autonomy to invest in rural infrastructure, human capital, and job creation programs. The VFP empowered the village leadership and community to make decisions on their development priorities and to invest in these priorities. It also had the potential to create alternative labour market opportunities, particularly for rural women which could have potential flow-on effects on household welfare and reduce poverty in rural areas.

Rural poverty rates (13%) in Indonesia are nearly double that in urban areas (7%) in 2019 [11]. About one-third of the Indonesian population was employed in agriculture 2019, with women accounting for 26.3 percent of all workers in the agricultural sector, approximately 34.54 million people [12]. From a policy perspective, the critical role of female labour force participation in enhancing human capital, demographic outcomes and economic performance is well-established in the literature [13]. Furthermore, reducing gender inequality in labour market participation was identified as an important means to address agricultural productivity, food security and rural economic development [14]. For example, transferring assets and skills to poor rural Bangladeshi women enabled them to increase their aggregate labour supply and earnings, which in turn reduced household poverty [15]. Similarly, providing alternative job opportunities for rural women improved the welfare of households in a study from Africa [16]. Studies from India have shown that improving young women’s labour market returns delayed their marriage and had flow on effects on the enrolment of girls in school [17,18].

Although the Indonesian economy has grown over the last two decades, as in other developing countries, the labour market participation of females has been relatively stagnant and significantly lags male participation rates [19]. Only about 50 percent of working-age women are active in the labour force compared to 82 percent of working-age men. Women also have a significantly higher probability of working in the informal sector compared to men and account for the bulk of self-employed and unpaid family workers [20]. Furthermore, young women in rural areas had reduced their labour force participation by opting out of informal/unpaid employment. There is also evidence of a U-shaped relationship between female labour force participation and education and wealth, suggesting that despite large gains in income and educational attainment, the growth in female labour force participation at the top end of the income and education distribution has largely been offset by losses at the bottom end of the distribution [19]. Other studies have attributed the low labour market participation of Indonesian women to socio-economic status [21], education [22], and social norms [23]. The VFP could then be viewed as an important step to reduce the socio-economic disparities between rural and urban areas.

This paper takes advantage of a unique household data set with national coverage (for the years 2012, 2013, 2017, 2019) representing a period before and after the introduction of the VFP. The research design focuses on a comparison of changes in officially designated rural villages (*desa*) that were recipients of the VFP, compared to similar urban villages that did not receive the program. Our *Difference-in-Differences* (DiD) estimation methods allows us to estimate the impact of the VFP among rural villages (*desa*) (*Village Fund* recipients- Treatment group) with urban villages (*Kelurahan*, our Control group), before and after the implementation of the program. Using a sample of 1361 villages, previous research shows that the implementation of the VFP was associated with an increase in the number of village-owned enterprises in recipient villages [24].

Specifically, we investigate the following research questions: (i) was the VFP associated with poverty reduction among rural households in recipient villages? (ii) Was there any narrowing in rural-urban labour force participation rates since the implementation of the VFP? (iii) Was an increase in labour force participation (particularly among women), the channel through which rural welfare improved? and finally (iv) whether there were sectoral changes in the composition of female employment in rural villages after the implementation of the VFP.

To the best of our knowledge our paper is the first nationally representative evaluation of the VFP on rural poverty and female labour force participation. Our results show that the VFP was associated with an improvement of labour force participation, particularly in off-farm activities. We identify this as the mechanism through which the implementation of the VFP led to improvements in rural welfare. We further show after the implementation of the VFP program, there was an increase in weekly working hours in rural areas by about ten percentage points. More interestingly, there was an increase in female labour force participation, and the increase in weekly working hours was higher relative to male working hours in rural areas. Our results also show that with the exception of agriculture, there was an increase in female labour force participation across all sectors. These findings suggest that the VFP has been successful in transforming female employment in rural areas away from the agriculture sector.

## 2. Background

### 2.1 Dana desa program (village fund program- VFP)

In 2014, the Indonesian Government embarked upon major reforms by shifting the paradigm of rural development from using a predominantly top-down approach to using a mixed approach which accorded a central role to villages. This reform marked by the enactment of Law No. 6 in 2014, gave larger authority to rural village leadership and provided stronger financial support. The objective was to improve the quality of life and welfare of village residents. The Village-focused reforms were carried out in the context of reducing the development gaps and socio-economic disparities between rural and urban areas.

In Indonesia, there are two types of villages: *desa* (rural) and *kelurahan* (urban). In a *desa*, the village head is elected directly by villagers, while in *kelurahan*, the village head is appointed by the district mayor. Since its independence from Dutch rule, the Government of Indonesia (GoI) has made several efforts to reform village governance. In January 2014, the GoI issued Law No. 6/2014 for *Desa* (the *Village Law* or *Undang Undang Desa* (UUDesa)), with the express intention of addressing gaps in Indonesia’s decentralization paradigm, and of enshrining the principles of community-driven development (CDD) as core principles in formal governance systems [25]. The GoI, through the UUDesa, aimed to improve autonomy in rural villages (*desa*), including providing them with greater budget allocations and improved governance arrangements. Under this new reform, the central government recognized the sovereignty and autonomy of the country’s rural villages. It confirmed their right to prioritize and manage village-level development based on principles of self-governing communities and of local self-government.

In particular, in 2014 the GoI implemented one of the most extensive village-level programs in the world, known as *Program Dana Desa* (or Village Fund Program (VFP). Through this program, the GoI sought to improve village-level governance by providing local leaders from around 75,000 rural villages with greater autonomy and fiscal capacity to select and invest in their development priorities. These reforms were supplemented with fiscal transfers of approximately US$ 7.3 billion (Rp 100 trillion) yearly to rural villages. Through providing rural villages with greater autonomy and fiscal capacity, the GoI aimed to reduce rural poverty and inequality. They further sought to improve the quality of VFP spending [26].

The VFP sought to reduce the inequities in rural-urban development, through fiscal transfers and fostering better governance through empowering the village leadership and community make decisions on their development priorities and to invest in these priorities. There are several aspects of the program which could potentially improve female labour force participation.

In the three years since the implementation of the *Dana Desa* program (2014–2016), significant resources were disbursed for the provision of drainage, irrigation, and construction of roads. As reported in Table 1, nearly 250,000 new projects were implemented between 2014 to 2016 using the Village Fund program. More importantly, minimum of 30% of the VFP fund should be disbursed to create the employment through cash for work program (or Padat Karya Tunai Desa) [27].

**Table 1:** Major projects built using *Dana Desa* Program, 2014–2016.

Rank	Major Project	Number of Project	Measure
1	Drainage and irrigation	103,405	units
2	Village Road	95,200	KMs
3	Well	19,485	units
4	Clean Water	22.616	units
5	Early childhood education	14.957	units
6	Village health centre	4004	units
7	Village market	3106	units
8	Water Pond	1338	units
9	Bridge	914	units

*Source*: The Ministry of Village, Development of Disadvantaged Regions and Transmigration.

Although the specific aim of the VFP was to reduce the gap between rural and urban development, local communities (including women) were granted better access to development resources. There are two aspects of the program which could potentially improve labour force participation, especially among women. Firstly, the Law explicitly acknowledges the role and position of women, thereby attempting to foster greater socio-economic empowerment for rural women [20]. Secondly, the Law stipulates the participation of women in all levels of development planning and implementation. Furthermore, increases in budget allocation for village development potentially opens new opportunities for women’s empowerment programs.

The requirement for budget transparency, included women’s involvement in the monitoring and evaluation of local development. Under these circumstances, an increase in female labour force participation could potentially improve the household’s economic status. By increasing female labour force participation and creating employment opportunities in more productive sectors, the VFP has the potential to reduce rural poverty and improve rural welfare.

The VFP is part of a long series of rural reforms by the GoI to address the significant inequities in rural-urban development. Over the period 1994 to 1997, the Government of Indonesia (GoI) implemented the first targeted poverty alleviation program, namely *Inpres Desa Tertinggal* (IDT or Left Behind Village Program), through increasing capital of the poor households in selected villages [28]. There is ambiguity in the literature on the success of this program in reducing poverty and improving labour supply. Previous research shows that the IDT program had no significant effects on labour supply and household expenditure [29]. On the other hand, some find that the IDT program was successful in reducing poverty among poor households [30,31], while other studies show that in wealthier and more unequal villages, more resources were provided to relatively wealthy households in the village [32]. However, unlike the VFP which is available to all Indonesian rural villages, IDT was only implemented in selected villages (around one-third, around 20,000).

Another strand of literature has examined the effectiveness of poverty targetting of specific social programs. This literature includes studies [33,34] using Randomised Control Trial (RCT) methods in a select group of villages, and nationally representative studies that have, for example the *Askeskin* and *Jamkesmas* programs [35,36] or the *Raskin* program [37,38]. Others evaluate the complementarity of various social welfare programs, and examine whether they reached the intended beneficiaries [39,40]. However, the focus of this literature has been on the targetting of social welfare programs for poor households, and not on a nation-wide reform of governance and fiscal transfers to rural villages.

Our paper focuses on a large nationally representative program of fiscal transfers and devolution of decision-making to rural villages, and its subsequent impact of rural-urban inequalities, through improvements in rural female labour force participation and a reduction in rural poverty.

## 3. Data and research methods

The data for our analysis come from four waves of the National Socioeconomic Survey (SUSENAS) survey (covering waves 2012, 2013, 2017 and 2019), representing two periods before and two periods after the implementation of the VFP. The SUSENAS is an annual cross-sectional, nationally representative dataset, initiated in 1963–1964 and fielded once every year or two since then. The SUSENAS survey is administered by the Indonesia Statistical Office (BPS -*Badan Pusat Statistik*) and includes data on household characteristics such as their socio-economic, demographic characteristics, as well as detailed information on their access to various social protection programs. We use the SUSENAS to construct our outcome and control variables at the household and individual levels.

Note that the data used in the analyses are anonymized secondary data that are publicly available upon registration and payment of fees from the Statistics Indonesia (BPS website (https://www.bps.go.id/). All Ethics approvals for the data collection were obtained from BPS.

In Table 1 we present descriptive statistics from SUSENAS datasets for the key variables used in the empirical analysis, where we compare differences between households living in rural and urban villages. We observe that during the study period, around 14.1 percent are classified as poor using the national poverty line, while only 7.2 percent of urban households are regarded as poor. Not surprisingly, a majority of rural household heads are employed in the agriculture sector (61.6 percent), compared to less than 20 percent among households in urban villages. We also observe that a significantly higher proportion of rural villages are beneficiaries of social welfare programs, relative to households in urban villages. Not surprisingly, urban household heads are significantly better educated with 32.5 percent having Senior High level of education, compared to 15.4 percent among rural households.

The summary statistics of key variables used in the household level analysis disaggregated by periods before and after the implementation of the VFP are presented in Tables A1 and A2 in the S1 Appendix. Prior to the implementation of the VFP, based on national poverty lines, the proportion of poor people was 7.6 percent higher in rural areas compared to urban areas. After the implementation of the VFP, the poverty rate in rural areas declined significantly to 12.5 percent or 6.2 percent lower than in urban areas.

Table 2 presents the summary statistics of our individual-level variables. According to Table 2, labour market participation as measured by the number of hours worked among workers aged 15 to 65 is lower in rural areas than among urban residents. Over the period of our study, the log of weekly working hours in urban and rural areas are 3.643 and 3.425, respectively. Similarly, the weekly working hours for rural females in the rural area is also lower compared to women in urban areas. In terms of education, urban have higher education levels, relative to rural residents. For instance, just 17.9 percent of individuals in rural areas have completed Senior High level of schooling, while this figure is around 29.8 percent in urban areas. We similarly see higher completion rates for university education among urban residents. Further, we can see that approximately 44 percent of rural residents are employed in the agriculture sector.

**Table 2:** Summary statistics of key and control variables for individual level analysis.

	Urban		Rural		Diff.	SE
	Mean	SD	Mean	SD	[3]—[1]	
	[1]	[2]	[3]	[4]	[5]	[6]
Log (working hours per week)	3.727	0.516	3.551	0.542	-0.176***	[0.001]
Log (female working hours per week)	3.643	0.572	3.425	0.587	-0.218***	[0.001]
Age	39.165	16.106	39.670	16.409	0.504***	[0.018]
Gender: male	0.491	0.500	0.496	0.500	0.005***	[0.001]
Marital status	0.641	0.480	0.690	0.463	0.049***	[0.001]
The Highest diploma:						
Elementary	0.203	0.402	0.316	0.465	0.113***	[0.000]
Junior high	0.160	0.366	0.154	0.361	-0.006***	[0.000]
Senior high	0.298	0.457	0.179	0.384	-0.119***	[0.000]
University	0.177	0.381	0.078	0.268	-0.098***	[0.000]
Economic sector:						
Agriculture	0.116	0.321	0.440	0.496	0.324***	[0.000]
Mining & Quarrying	0.045	0.206	0.028	0.166	-0.016***	[0.000]
Processing Industry	0.065	0.247	0.035	0.183	-0.031***	[0.000]
Electricity & gas	0.012	0.110	0.004	0.061	-0.009***	[0.000]
Construction/building	0.024	0.153	0.015	0.122	-0.009***	[0.000]
Trading	0.093	0.290	0.045	0.207	-0.048***	[0.000]
Hotel & Restaurant	0.047	0.211	0.022	0.148	-0.024***	[0.000]
ICT	0.043	0.204	0.018	0.134	-0.025***	[0.000]
Finance & insurance	0.019	0.136	0.007	0.081	-0.012***	[0.000]
Educational services	0.029	0.169	0.018	0.131	-0.012***	[0.000]
Health services	0.009	0.095	0.003	0.057	-0.006***	[0.000]
Public services	0.061	0.239	0.021	0.143	-0.040***	[0.000]
Others	0.018	0.131	0.007	0.081	-0.011***	[0.000]
Head of HHD working	0.845	0.362	0.919	0.273	0.074***	[0.000]
Head economic sectors:						
Agriculture	0.196	0.397	0.626	0.484	0.430***	[0.000]
Industry	0.178	0.382	0.095	0.294	-0.082***	[0.000]
Services	0.447	0.497	0.188	0.391	-0.259***	[0.001]
Others	0.023	0.151	0.010	0.098	-0.014***	[0.000]
Dependency Ratio	0.525	0.559	0.622	0.619	0.097***	[0.001]
Member age below 4 yo	0.339	0.579	0.371	0.597	0.032***	[0.001]
Member age below 5–12 yo	0.937	1.016	1.066	1.094	0.129***	[0.001]
Number of observations	854921		1237602			

*Notes*: This table presents the summary statistics of key and control variables for individual level analysis from SUSENAS surveys. *,**, and *** represent statistical significance at 10, 5, and 1 percent, respectively.

**Fig 1:**
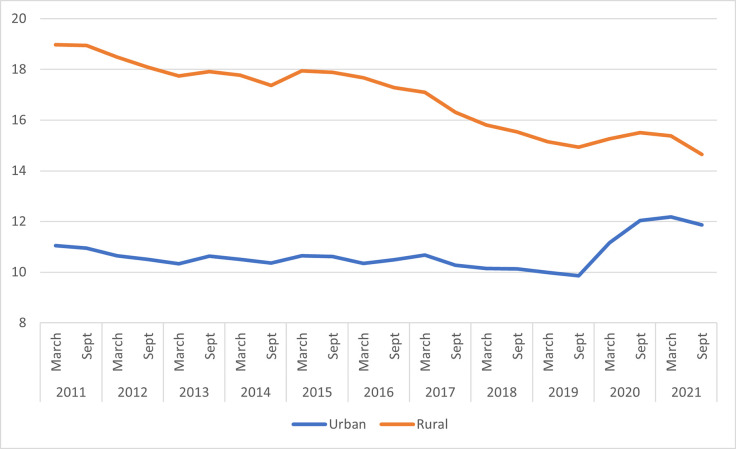
Rural and urban poverty: 2011–2021. Source: BPS (2021).

Fig 1 below shows a narrowing of the gap between rural and urban poverty between 2011 and 2021. This decline rural poverty is much more discernible after the introduction of the VFP.

In Tables A3 and A4 in S1 Appendix, we also present the summary statistics for variables used in our individual-level analysis, disaggregated by before and after the implementation of the VFP. Prior to the implementation of the VFP, labour market participation in rural areas was 21.4 percentage points lower than in urban areas. After the implementation of the VFP, however, the rural-urban differences in labour market participation narrows to about 14.1 percentage points. Similarly, for female labour market participation, we observe that the rural-urban gap in labour market participation shrinks after the implementation of the VFP from 26.5 to 17.1 percentage points, respectively. This is also consistent with the narrowing of poverty between rural and urban areas.

### 3.1. Estimation strategy

To estimate the impact of the Dana Desa program on rural poverty and female labour force participation we use *Difference-in-Differences* (DiD) methods. Specifically, we compare our outcomes of interest (poverty and female labour force participation) among rural villages (*Desa*) (*Village Fund* recipients- Treatment group) with urban villages (*Kelurahan*, our Control group), before and after the implementation of the program. Note that the DiD estimator is defined as the difference in average outcomes in the treatment group before and after treatment *minus* the difference in average outcome in the control group before and after treatment. The main drawback of using simple before and after the treatment is that the estimator will be biased if a time-trend exists in the outcome variables. In this case, we will confound the time trend as being part of the treatment effect. Given that the VFP was implemented in all rural villages, it is appropriate to compare if the rural-urban differential in poverty declined after the implementation of the VFP.

As previously indicated, poverty is measured using the national poverty line produced by the BPS. Our outcome variable for household poverty status is a binary variable that takes on a value of 1 if the household’s per capita consumption expenditure is below the national poverty line, 0 otherwise. Labour force participation, including the female labour, is defined as the number of working hours per week.

Our *DiD* specification is given by:

$$Y_{ivdt}=\alpha +{\beta V}_{v}+{\pi Post}_{t}+{\delta (V}_{v}.\;{Post}_{t})+D_{d}+X_{i}+\varepsilon_{i}$$ (1)

Where *Y_ivt_* refers to our outcome variables, namely poverty and labour force participation in household *i*, village *v*, district *d*, and in the period *t*. *V_v_* is a dummy variable indicating a rural village (*Desa*). The term *Post_t_* refers to a dummy variable for the period after the implementation of the *Village Fund* program. Our parameter of interest is *δ* which captures the effect of the program on our variables of interest. We also include district fixed effects (*D*) to control for unobserved heterogeneity within districts and a vector of control variables as presented in Tables 1 and 2, *X_i_*. *ε_i_* is the error term.

Ideally, we could include fixed effects at the lower administrative levels (such as village or sub-district). However, we are unable to do so since in the SUSENAS survey, the BPS has not provided the code for administrative levels lower than districts since 2012. All standard errors are clustered at the household level.

## 4. Results

### 4.1. Village fund program and rural poverty

The first aim of this paper is to examine the effect of the VFP on rural poverty. Accordingly, we examine whether the implementation of the VFP improved welfare in rural households, measured using household poverty. We estimate a DiD model as in Eq (1) with two dependent variables, household poverty status for the full sample and for the sample of poor households (as a robustness check). For the first variable, we test whether the implementation of the VFP reduced poverty among rural households.

As reported in Columns [1] to [3] of Table 3, without controlling for any other variables, the implementation of the VFP decreased poverty among households in rural villages by about 1.7 percentage points compared to residents of urban villages. When we include controls for whether the household received social welfare programs, the size of the effect decreases slightly to 1.4 percentage points (Table 3, Column [2]).

**Table 3:** The impact of the VFP on rural poverty.

	Dependent variable:					
	Pr (Poverty = 1)			Log (Per capita Consumption expenditure of the Poor)		
	[1]	[2]	[3]	[4]	[5]	[6]
Post	-0.018	0.001	-0.005	0.334	0.329	0.335
	(0.001)***	(0.001)	(0.001)***	(0.002)***	(0.002)***	(0.002)***
*Desa*	0.051	0.032	0.019	-0.067	-0.066	-0.053
	(0.001)***	(0.001)***	(0.001)***	(0.002)***	(0.002)***	(0.002)***
Post X *Desa*	-0.017	-0.014	-0.009	0.031	0.033	0.026
	(0.001)***	(0.001)***	(0.001)***	(0.002)***	(0.002)***	(0.002)***
District Fixed Effect	Y	Y	Y	Y	Y	Y
Control variables include:						
Social Protection	N	Y	Y	N	Y	Y
Head	N	N	Y	N	N	Y
Household	N	N	Y	N	N	Y
Number of households	1,183,123	1,183,123	1,183,123	131,871	131,871	131,871
*R* ^*2*^	0.074	0.091	0.162	0.620	0.621	0.641

*Notes*: *The* outcome variables in Column [1] to [3] are binary variables equals to 1 if poor and 0 otherwise, while in Column [4] to [5] are the log per capita consumption of the poor households. Control variables included in the model are presented in the Table 1. All standard errors are clustered at the household level. *,**, and *** represent statistical significance at 10, 5, and 1 percent, respectively.

To test the robustness of our results, we also estimate the effect of the VFP on poverty in the sub-sample of poor households. For this exercise, we estimate Eq (1) only for the sub sample of poor households. As reported in Columns [4] to [6] of Table 3, the implementation of the village fund program increased the per capita consumption expenditure of poor households in rural areas compared to those in urban areas by about 3 percentage points. For instance, without including any other control variables, the implementation of the VFP increases the household per capita consumption expenditure by about 3.1 percentage points (Column [4]). The results do not significantly change when we control for household head and household information, and whether the household received social welfare benefits.

### 4.2. Did the VFP improve rural welfare?

As outlined above, the implementation of the village fund program reduced rural poverty in general, and more specially improved welfare in the sub-sample of poor rural households. To identify the groups which may have benefited most from the program, we estimate the effect of the village fund program on the per capita consumption expenditure of agriculture and non-agriculture households. Agriculture households are those where the household head works in the agriculture sector. We estimate DiD models as in Eq (1) separately for agricultural and non-agricultural households using the log of household per capita consumption expenditure as the dependent variable. The results presented in Table 4 show that the implementation of the VFP increased per capita consumption among agricultural households in rural villages. As reported in Columns [1] to [3], the implementation of the program was followed by an increase in per capita consumption expenditure of agriculture households by about 5 to 7 percentage points compared to households in urban villages. While there was an increase in per capita consumption expenditure among non-agricultural households, the size of the effect was relatively smaller, about 2 to 6 percentage points.

**Table 4:** The impact of the VFP on rural welfare.

	Dependent variable: Log (Percapita Consumption expenditure)					
	Agricultural Households			Non- Agricultural Households		
	[1]	[2]	[3]	[4]	[5]	[6]
Post	0.458	0.399	0.409	0.450	0.351	0.372
	(0.004)***	(0.004)***	(0.003)***	(0.002)***	(0.002)***	(0.002)***
*Desa*	-0.101	-0.085	-0.051	-0.217	-0.116	-0.049
	(0.003)***	(0.003)***	(0.003)***	(0.002)***	(0.002)***	(0.002)***
Post X *Desa*	0.051	0.062	0.05	0.063	0.026	0.022
	(0.004)***	(0.004)***	(0.004)***	(0.003)***	(0.003)***	(0.003)***
District Fixed Effect	Y	Y	Y	Y	Y	Y
Control variables include:						
Social Protection	N	Y	Y	N	Y	Y
Head	N	N	Y	N	N	Y
Household	N	N	Y	N	N	Y
Number of households	514,341	514,341	514,341	668,782	668,782	668,782
*R* ^*2*^	0.319	0.344	0.507	0.268	0.348	0.532

*Notes*: The outcome variable is log percapita consumption. Agriculture households in Column [1] to [3] refers to households which the head worked in the agriculture sector. Column [4] to [5] represents estimations from non- agriculture households. Control variables included in the model are presented in the Table 1. All standard errors are clustered at the household level. *,**, and *** represent statistical significance at 10, 5, and 1 percent, respectively.

The key finding from Table 4 is that the implementation of the VFP is statistically significant and positively associated with an improvement in agricultural household’s per capita consumption expenditure, our proxy for poverty. In the next section, we investigate the factors that may be driving this reduction in poverty.

### 4.3. Role of female labour force participation in reducing household poverty

Having established that the implementation of the VFP increased household per capita consumption expenditure and reduced poverty in rural villages, in this section, we investigate the potential channels that may be potentially driving these changes. Previous research has already identified the important role of female economic participation in improving outcomes for their households [41–44].

In the next section, we examine if female labour force participation was the channel through which VFP reduced poverty and improved the welfare in rural areas.

#### A. Labour market participation

To examine the impact of the VFP on labour force participation, we estimate Eq (1) with using weekly log of working hours as our dependent variable. We test whether there was a change in working hours after the implementation of the VFP, and whether this differs between males and females. As reported in Table 5, the implementation of the village fund program was associated with higher hours of labour market participation in rural villages. For instance, from Panel A of Table 5, we observe that after the implementation of the VFP, there was an increase of about 10 percentage points in weekly working hours among rural residents relative to urban residents. These results do not change much when we include controls for education and other individual characteristics such as respondent’s age and marital status. However, when we include the sector of employment, the size of the effect decreases to 6.5 percentage points (as reported in Column 4, Panel A of Table 5). In the Columns [5] and [6] of Table 5, we also include the control variables related to the head of the household (such as whether the household head is working and their sector of employment) and the number of the family member aged below 4 and 12 years old.

**Table 5:** The impact of VFP on rural labour force participation.

	Dependent variable: Log (Weekly working Hours)					
	[1]	[2]	[3]	[4]	[5]	[6]
Panel A: Male + Female						
Post	0.071	0.074	0.078	0.110	0.111	0.111
	(0.001)***	(0.001)***	(0.001)***	(0.002)***	(0.002)***	(0.002)***
*Desa*	-0.182	-0.174	-0.162	-0.083	-0.084	-0.084
	(0.001)***	(0.001)***	(0.001)***	(0.001)***	(0.001)***	(0.001)***
Post X *Desa*	0.106	0.102	0.094	0.064	0.063	0.063
	(0.002)***	(0.002)***	(0.002)***	(0.002)***	(0.002)***	(0.002)***
Number of observations	2,073,561	2,073,561	2,073,561	2,073,561	2,073,561	2,073,561
*R* ^*2*^	0.059	0.104	0.106	0.138	0.138	0.138
Panel B: Male						
Post	0.066	0.070	0.075	0.103	0.106	0.106
	(0.002)***	(0.002)***	(0.002)***	(0.002)***	(0.002)***	(0.002)***
*Desa*	-0.149	-0.140	-0.134	-0.076	-0.076	-0.076
	(0.001)***	(0.001)***	(0.001)***	(0.002)***	(0.002)***	(0.002)***
Post X *Desa*	0.087	0.085	0.079	0.056	0.054	0.054
	(0.002)***	(0.002)***	(0.002)***	(0.002)***	(0.002)***	(0.002)***
Number of observations	1,267,355	1,267,355	1,267,355	1,267,355	1,267,355	1,267,355
*R* ^*2*^	0.058	0.092	0.093	0.116	0.117	0.117
Panel C: Female						
Post	0.082	0.084	0.087	0.137	0.138	0.137
	(0.002)***	(0.002)***	(0.002)***	(0.003)***	(0.003)***	(0.003)***
*Desa*	-0.231	-0.220	-0.200	-0.080	-0.080	-0.078
	(0.002)***	(0.002)***	(0.002)***	(0.002)***	(0.002)***	(0.002)***
Post X *Desa*	0.127	0.126	0.114	0.064	0.063	0.062
	(0.003)***	(0.003)***	(0.003)***	(0.003)***	(0.003)***	(0.003)***
Number of observations	806,206	806,206	806,206	806,206	806,206	806,206
*R* ^*2*^	0.069	0.082	0.085	0.134	0.135	0.135
District Fixed Effect	Y	Y	Y	Y	Y	Y
Control variables include:						
Personal characteristics	N	Y	Y	Y	Y	Y
Education	N	N	Y	Y	Y	Y
Economic Sectors	N	N	N	Y	Y	Y
Head of the households	N	N	N	N	Y	Y
Member of household	N	N	N	N	N	Y

*Notes*: This table presents the impact of the VFP on village labour market. The outcome variable is log working hours in a week. Panel A, we estimate for the whole sample combining male and female labour force, while in Panel B and C we estimate separately of subsample male and female labour force, respectively. Control variables included in the model are presented in the Tables 1 and 2. All standard errors are clustered at the household level. *,**, and *** represent statistical significance at 10, 5, and 1 percent, respectively.

Notably, although both rural male and female labour force participation increased since the implementation of the VFP, we observe a relatively higher increase in female labour force participation (Panel B and C of Table 5). In particular, as reported in Column [1] of Panel B and C, after the implementation of the village fund program, there is an increase of about 9.5 and 13.2 percentage points in the working hours of males and females in rural villages compared to their urban village counterparts, respectively. To corroborate these results, we also conduct estimations to verify whether the implementation of VFP has an impact on unemployment. These results are shown in Table A5 in S1 Appendix. Overall, having controlled for individual and household characteristics, our estimates show that the implementation of the VFP reduced unemployment in rural villages by about 2 percentage points.

Summing up, the implementation of the VFP has increased the labour force participation in rural areas. This may be one potential mechanism through which the VFP was associated with an increase in the per capita consumption expenditure among households in rural villages. In the next section, we further investigate the specific sectors in which rural female labour participation increased.

#### B. Sectoral mobility in female labour force participation

In this section, we further investigate the sectors where female labour force participation increased. Using the same specification as in Eq (1), we estimate a DiD model using sectoral female labour force participation as our dependent variable. In this exercise, we use an indicator variable which takes on a value of 1 if the female is working in a particular sector and zero otherwise. In Panel A, for example, the dependent variable takes on a value of 1 if the female is employed in the agriculture sector. In Panels B, C, and D, the dependent variable is equal to 1 if the female is employed in industry, services, other sectors, respectively.

Overall, as shown in Table 6, the implementation of the VFP is associated with rural female labour moving to the services sector from other sectors. For instance, as shown in Panel (A) of Table 6, since the introduction of the VFH, there has been a reduction of female labour force participation in the agriculture sector by about 10 percentage points in rural villages. Concurrently, the industrial and other economic sectors in rural villages have experienced a decrease in female labour force participation by about 1.1 and 1 percentage points, respectively. However, as reported in Panel (C) of Table 6, the services sector experienced an increase of about 9.9 percentage points after the implementation of the VFP.

**Table 6:** The impact of the VFP on rural female labour sectoral mobility.

	Female Labour Sectoral Mobility		
	[1]	[2]	[3]
*Panel A*: *Agriculture*			
Post X *Desa*	-0.096	-0.098	-0.086
	(0.001)***	(0.001)***	(0.001)***
*R* ^*2*^	0.143	0.208	0.226
*Panel B*: *Industry*			
Post X *Desa*	-0.011	-0.011	-0.012
	(0.001)***	(0.001)***	(0.001)***
*R* ^*2*^	0.021	0.033	0.033
*Panel C*: *Services*			
Post X *Desa*	0.099	0.097	0.095
	(0.001)***	(0.001)***	(0.001)***
*R* ^*2*^	0.058	0.105	0.120
*Panel D*: *Other sectors*			
Post X *Desa*	-0.010	-0.010	-0.010
	(0.000)***	(0.000)***	(0.000)***
*R* ^*2*^	0.008	0.010	0.011
Observations	1,852,936	1,852,936	1,852,936
Controlled with:			
Personal information	N	Y	Y
Education	N	N	Y

*Notes*: This table presents the impact of the VFP on female labour force participation across economic sectors. The dependent variable is binary variable equal 1 if the female labour is working in a sector, 0 otherwise. Control variables included in the model are personal information such as age, marital status, and level of education. All standard errors are clustered at the household level. *,**, and *** represent statistical significance at 10, 5, and 1 percent, respectively.

To sum up, we find that the implementation of the VFP has transformed female labour force in rural areas away from the agriculture sector. More specifically, due to the implementation of the VFP, the number of working hours in a week in other sectors, particularly in the service sector has increased significantly.

## 5. Conclusion

Globally, rural residents continue to account for the bulk of poverty in developing countries. In 2014, Indonesia embarked on an ambitious nation-wide rural development program (called the *Dana Desa* program (Village Fund Program or VFP), that provided greater autonomy to rural village leadership and communities to invest in their development priorities. Using nationally representative data, this paper sought to evaluate the effectiveness of this recently introduced program in reducing rural poverty and improving labour force participation, particularly among women. Using *Difference-in-Differences* (DiD) empirical methods, we compared outcomes among recipient rural villages and non-recipient urban villages.

Our main results suggest that the implementation of the VFP reduced rural poverty and improved the welfare of people who reside in the rural recipient villages. More specifically, the implementation of the VFP reduced rural poverty by about 1.7 percentage points compared to urban villages. Our results also suggest that the implementation of the program increased the per capita consumption expenditure of poor households in recipient villages by about 3.1 percentage points compared to their counterparts in urban areas. We further show that agricultural households in rural villages benefitted relatively more than non-agricultural households.

We investigate whether patterns of labour force participation can help explain how the VFP was associated with lower poverty in rural areas. Our results show that there is an increase of about ten percentage points in weekly working hours in rural villages compared to the urban villages after the implementation of the VFP. More interestingly, there was a relatively higher increase in working hours among females in rural areas relative to their male counterparts. In particular, there was also a shift away from agriculture towards the service sector.

We acknowledge a limitation of the study. Ideally, if we could match the PODES and SUSENAS datasets, we could have more information about the village, such as the level of development, population size, infrastructure readiness, and other important village-level information. This would have allowed us to find the perfect counterfactual from the rural villages.

However, since 2012, the BPS does not publish the village codes for the SUSENAS survey. Given this data limitation, we could only use information on the rural/urban village classification in the SUSENAS to evaluate the effectiveness of the VFP program. For these reasons, DiD is the best possible empirical strategy that can be used.

Despite these limitations, our study shows that in the short period that it has been operational, the ambitious VFP was successful in reducing poverty and improving rural female labour force participation. It was also effective in narrowing the rural-urban gaps in poverty and labour force participation.

## Supporting information

S1 Appendix (DOCX)
