# Supplementary material for: Rural poverty and labour force participation: Evidence from Indonesia’s Village fund program
Source: PLoS One. 2023 Jun 21;18(6):e0283041. doi: 10.1371/journal.pone.0283041 (PMC10284375; doi:10.1371/journal.pone.0283041)
Supplement: S1 Appendix — (DOCX) [file pone.0283041.s001.docx]

**Supplementary Appendix**

**Table A1** Descriptive statistics of variables at household level – Before the VFP

|  | Urban | | Rural | | Diff. | SE |
| --- | --- | --- | --- | --- | --- | --- |
|  | Mean | SD | Mean | SD | [3] - [1] |  |
|  | [1] | [2] | [3] | [4] | [5] | [6] |
| Poor | 0.081 | 0.273 | 0.157 | 0.364 | 0.076*** | [0.001] |
| Log (percapita consumption) | 13.500 | 0.713 | 13.063 | 0.581 | -0.437*** | [0.002] |
| Social protection beneficiary: |  |  |  |  |  |  |
| Health program | 0.225 | 0.418 | 0.325 | 0.468 | 0.100*** | [0.001] |
| Raskin/BPNT program | 0.346 | 0.476 | 0.585 | 0.493 | 0.239*** | [0.001] |
| The Highest diploma obtained by head: | |  |  |  |  |  |
| Elementary | 0.226 | 0.418 | 0.342 | 0.475 | 0.117*** | [0.001] |
| Junior high | 0.157 | 0.363 | 0.142 | 0.349 | -0.014*** | [0.001] |
| Senior high | 0.323 | 0.467 | 0.138 | 0.345 | -0.185*** | [0.001] |
| University | 0.128 | 0.334 | 0.033 | 0.180 | -0.095*** | [0.001] |
| Whether head works | 0.848 | 0.359 | 0.915 | 0.279 | 0.067*** | [0.001] |
| Economic sector of the head: |  |  |  |  |  |  |
| Agriculture | 0.140 | 0.347 | 0.599 | 0.490 | 0.459*** | [0.001] |
| Mining & Quarrying | 0.019 | 0.136 | 0.023 | 0.149 | 0.004*** | [0.000] |
| Processing Industry | 0.089 | 0.284 | 0.042 | 0.201 | -0.047*** | [0.001] |
| Electricity & gas | 0.005 | 0.073 | 0.001 | 0.039 | -0.004*** | [0.000] |
| Construction/building | 0.087 | 0.282 | 0.055 | 0.229 | -0.032*** | [0.001] |
| Trading | 0.178 | 0.383 | 0.071 | 0.257 | -0.107*** | [0.001] |
| Hotel & Restaurant | 0.021 | 0.144 | 0.004 | 0.064 | -0.017*** | [0.000] |
| ICT | 0.077 | 0.266 | 0.030 | 0.172 | -0.046*** | [0.001] |
| Finance & insurance | 0.012 | 0.108 | 0.002 | 0.040 | -0.010*** | [0.000] |
| Educational services | 0.034 | 0.181 | 0.020 | 0.141 | -0.014*** | [0.000] |
| Health services | 0.009 | 0.092 | 0.003 | 0.053 | -0.006*** | [0.000] |
| Public services | 0.166 | 0.372 | 0.059 | 0.235 | -0.108*** | [0.001] |
| Others | 0.011 | 0.106 | 0.005 | 0.072 | -0.006*** | [0.000] |
| Household size | 3.869 | 1.732 | 3.877 | 1.732 | 0.008 | [0.005] |
| Percentage of the household working in: | |  |  |  |  |  |
| Agriculture | 0.070 | 0.182 | 0.345 | 0.318 | 0.276*** | [0.001] |
| Industry | 0.066 | 0.166 | 0.039 | 0.129 | -0.028*** | [0.000] |
| Services | 0.344 | 0.288 | 0.145 | 0.230 | -0.198*** | [0.001] |
| Other | 0.007 | 0.055 | 0.003 | 0.038 | -0.003*** | [0.000] |
| Dependency ratio | 0.603 | 0.615 | 0.715 | 0.683 | 0.112*** | [0.002] |
| # of member age below 4 | 0.331 | 0.563 | 0.359 | 0.584 | 0.029*** | [0.002] |
| # of member age 5-12 | 0.611 | 0.789 | 0.708 | 0.868 | 0.096*** | [0.002] |
| Number of households | 244183 | | 325993 | |  |  |

**Table A2** Descriptive statistics of key and control variables at household level – After the VFP

|  | Urban | | Rural | | Diff. | SE |
| --- | --- | --- | --- | --- | --- | --- |
|  | Mean | SD | Mean | SD | [3] - [1] |  |
|  | [1] | [2] | [3] | [4] | [5] | [6] |
| Poor | 0.063 | 0.244 | 0.125 | 0.331 | 0.062*** | [0.001] |
| Log (percapita consumption) | 13.927 | 0.694 | 13.570 | 0.592 | -0.358*** | [0.002] |
| Social protection beneficiary: |  |  |  |  |  |  |
| Health program | 0.099 | 0.298 | 0.171 | 0.376 | 0.072*** | [0.001] |
| Raskin/BPNT program | 0.180 | 0.384 | 0.384 | 0.486 | 0.204*** | [0.001] |
| The Highest diploma obtained by head: | |  |  |  |  |  |
| Elementary | 0.224 | 0.417 | 0.336 | 0.472 | 0.112*** | [0.001] |
| Junior high | 0.157 | 0.364 | 0.152 | 0.359 | -0.005*** | [0.001] |
| Senior high | 0.328 | 0.469 | 0.169 | 0.375 | -0.158*** | [0.001] |
| University | 0.135 | 0.342 | 0.046 | 0.210 | -0.089*** | [0.001] |
| Whether head works | 0.837 | 0.369 | 0.912 | 0.283 | 0.075*** | [0.001] |
| Economic sector of the head: |  |  |  |  |  |  |
| Agriculture | 0.236 | 0.425 | 0.631 | 0.482 | 0.395*** | [0.001] |
| Mining & Quarrying | 0.093 | 0.291 | 0.045 | 0.208 | -0.048*** | [0.001] |
| Processing Industry | 0.080 | 0.271 | 0.041 | 0.198 | -0.039*** | [0.001] |
| Electricity & gas | 0.023 | 0.151 | 0.006 | 0.078 | -0.017*** | [0.000] |
| Construction/building | 0.005 | 0.071 | 0.001 | 0.037 | -0.004*** | [0.000] |
| Trading | 0.057 | 0.231 | 0.035 | 0.183 | -0.022*** | [0.001] |
| Hotel & Restaurant | 0.084 | 0.278 | 0.039 | 0.194 | -0.045*** | [0.001] |
| ICT | 0.103 | 0.304 | 0.040 | 0.197 | -0.063*** | [0.001] |
| Finance & insurance | 0.022 | 0.146 | 0.011 | 0.102 | -0.011*** | [0.000] |
| Educational services | 0.017 | 0.128 | 0.012 | 0.109 | -0.005*** | [0.000] |
| Health services | 0.005 | 0.074 | 0.002 | 0.047 | -0.003*** | [0.000] |
| Public services | 0.074 | 0.262 | 0.033 | 0.180 | -0.041*** | [0.001] |
| Others | 0.038 | 0.190 | 0.015 | 0.121 | -0.023*** | [0.000] |
| Household size | 3.771 | 1.723 | 3.844 | 1.726 | 0.073*** | [0.004] |
| Percentage of the household working in: | |  |  |  |  |  |
| Agriculture | 0.117 | 0.219 | 0.343 | 0.310 | 0.226*** | [0.001] |
| Industry | 0.147 | 0.234 | 0.073 | 0.168 | -0.074*** | [0.001] |
| Services | 0.203 | 0.267 | 0.095 | 0.194 | -0.109*** | [0.001] |
| Other | 0.023 | 0.101 | 0.009 | 0.062 | -0.014*** | [0.000] |
| Dependency ratio | 0.571 | 0.608 | 0.669 | 0.659 | 0.098*** | [0.002] |
| # of member age below 4 | 0.306 | 0.550 | 0.338 | 0.568 | 0.032*** | [0.001] |
| # of member age 5-12 | 0.555 | 0.751 | 0.647 | 0.810 | 0.092*** | [0.002] |
| Number of households | 258385 | | 354562 | |  |  |

**Table A3** Descriptive statistics of key and control variables of individual level analysis – Before the VFP

|  | Urban | | Rural | | Diff. | SE |
| --- | --- | --- | --- | --- | --- | --- |
|  | Mean | SD | Mean | SD | [3] - [1] |  |
|  | [1] | [2] | [3] | [4] | [5] | [6] |
| Log (working hours) | 3.685 | 0.540 | 3.471 | 0.568 | -0.214*** | [0.001] |
| Log (female working hours) | 3.595 | 0.595 | 3.330 | 0.608 | -0.265*** | [0.002] |
| Age | 38.776 | 15.995 | 39.420 | 16.366 | 0.644*** | [0.026] |
| Gender: male | 0.491 | 0.500 | 0.496 | 0.500 | 0.006*** | [0.001] |
| Marrital status | 0.649 | 0.477 | 0.699 | 0.459 | 0.050*** | [0.001] |
| The Highest diploma: |  |  |  |  |  |  |
| Elementary | 0.208 | 0.406 | 0.329 | 0.470 | 0.121*** | [0.001] |
| Junior high | 0.210 | 0.408 | 0.194 | 0.396 | -0.016*** | [0.001] |
| Senior high | 0.328 | 0.469 | 0.153 | 0.360 | -0.175*** | [0.001] |
| University | 0.118 | 0.323 | 0.036 | 0.187 | -0.082*** | [0.000] |
| Economic sector: |  |  |  |  |  |  |
| Agriculture | 0.085 | 0.279 | 0.447 | 0.497 | 0.362*** | [0.001] |
| Mining & Quarrying | 0.010 | 0.100 | 0.014 | 0.119 | 0.004*** | [0.000] |
| Processing Industry | 0.075 | 0.264 | 0.039 | 0.192 | -0.037*** | [0.000] |
| Electricity & gas | 0.003 | 0.055 | 0.001 | 0.031 | -0.002*** | [0.000] |
| Construction/building | 0.045 | 0.208 | 0.031 | 0.173 | -0.015*** | [0.000] |
| Trading | 0.161 | 0.367 | 0.074 | 0.262 | -0.086*** | [0.001] |
| Hotel & Restaurant | 0.020 | 0.141 | 0.005 | 0.068 | -0.016*** | [0.000] |
| ICT | 0.040 | 0.195 | 0.018 | 0.132 | -0.022*** | [0.000] |
| Finance & insurance | 0.011 | 0.105 | 0.002 | 0.040 | -0.009*** | [0.000] |
| Educational services | 0.036 | 0.185 | 0.023 | 0.149 | -0.013*** | [0.000] |
| Health services | 0.011 | 0.104 | 0.004 | 0.063 | -0.007*** | [0.000] |
| Public services | 0.121 | 0.326 | 0.043 | 0.202 | -0.079*** | [0.000] |
| Others | 0.008 | 0.091 | 0.004 | 0.064 | -0.004*** | [0.000] |
| Head of HHD working | 0.850 | 0.357 | 0.920 | 0.271 | 0.070*** | [0.001] |
| Head working economic sectors: |  |  |  |  |  |  |
| Agriculture | 0.146 | 0.353 | 0.608 | 0.488 | 0.463*** | [0.001] |
| Industry | 0.103 | 0.303 | 0.063 | 0.243 | -0.040*** | [0.000] |
| Services | 0.591 | 0.492 | 0.244 | 0.429 | -0.347*** | [0.001] |
| Others | 0.011 | 0.104 | 0.005 | 0.071 | -0.006*** | [0.000] |
| Dependency Ratio | 0.539 | 0.562 | 0.645 | 0.633 | 0.106*** | [0.001] |
| # Member age below 4 yo | 0.349 | 0.583 | 0.380 | 0.603 | 0.031*** | [0.001] |
| # Member age below 5-12 yo | 0.973 | 1.026 | 1.106 | 1.115 | 0.133*** | [0.002] |
| Number of observations | 678913 | | 862005 | |  |  |

**Table A4** Descriptive statistics of key and control variables of individual level analysis – After the VFP

|  | Urban | | Rural | | Diff. | SE |
| --- | --- | --- | --- | --- | --- | --- |
|  | Mean | SD | Mean | SD | [3] - [1] |  |
|  | [1] | [2] | [3] | [4] | [5] | [6] |
| Log (working hours) | 3.766 | 0.489 | 3.625 | 0.506 | -0.141*** | [0.001] |
| Log (female working hours) | 3.688 | 0.546 | 3.515 | 0.552 | -0.173*** | [0.002] |
| Age | 39.534 | 16.201 | 39.895 | 16.445 | 0.360*** | [0.025] |
| Gender: male | 0.492 | 0.500 | 0.496 | 0.500 | 0.004*** | [0.001] |
| Marrital status | 0.634 | 0.482 | 0.682 | 0.466 | 0.048*** | [0.001] |
| The Highest diploma: |  |  |  |  |  |  |
| Elementary | 0.198 | 0.398 | 0.304 | 0.460 | 0.106*** | [0.001] |
| Junior high | 0.112 | 0.315 | 0.118 | 0.322 | 0.006*** | [0.000] |
| Senior high | 0.270 | 0.444 | 0.203 | 0.402 | -0.066*** | [0.001] |
| University | 0.232 | 0.422 | 0.116 | 0.320 | -0.116*** | [0.001] |
| Economic sector: |  |  |  |  |  |  |
| Agriculture | 0.146 | 0.353 | 0.434 | 0.496 | 0.287*** | [0.001] |
| Mining & Quarrying | 0.077 | 0.267 | 0.041 | 0.199 | -0.036*** | [0.000] |
| Processing Industry | 0.056 | 0.230 | 0.031 | 0.174 | -0.024*** | [0.000] |
| Electricity & gas | 0.021 | 0.144 | 0.006 | 0.079 | -0.015*** | [0.000] |
| Construction/building | 0.004 | 0.062 | 0.001 | 0.032 | -0.003*** | [0.000] |
| Trading | 0.028 | 0.166 | 0.018 | 0.134 | -0.010*** | [0.000] |
| Hotel & Restaurant | 0.071 | 0.258 | 0.039 | 0.193 | -0.033*** | [0.000] |
| ICT | 0.047 | 0.212 | 0.019 | 0.137 | -0.028*** | [0.000] |
| Finance & insurance | 0.026 | 0.160 | 0.011 | 0.105 | -0.015*** | [0.000] |
| Educational services | 0.023 | 0.151 | 0.013 | 0.112 | -0.011*** | [0.000] |
| Health services | 0.008 | 0.087 | 0.003 | 0.051 | -0.005*** | [0.000] |
| Public services | 0.003 | 0.057 | 0.001 | 0.032 | -0.002*** | [0.000] |
| Others | 0.026 | 0.160 | 0.009 | 0.093 | -0.018*** | [0.000] |
| Head of HHD working | 0.840 | 0.367 | 0.918 | 0.275 | 0.078*** | [0.001] |
| Head working economic sectors: |  |  |  |  |  |  |
| Agriculture | 0.244 | 0.430 | 0.642 | 0.480 | 0.397*** | [0.001] |
| Industry | 0.249 | 0.433 | 0.125 | 0.330 | -0.125*** | [0.001] |
| Services | 0.312 | 0.463 | 0.138 | 0.345 | -0.174*** | [0.001] |
| Others | 0.035 | 0.183 | 0.014 | 0.116 | -0.021*** | [0.000] |
| Dependency Ratio | 0.513 | 0.555 | 0.601 | 0.607 | 0.088*** | [0.001] |
| # Member age below 4 yo | 0.329 | 0.575 | 0.362 | 0.591 | 0.033*** | [0.001] |
| # Member age below 5-12 yo | 0.903 | 1.006 | 1.030 | 1.072 | 0.127*** | [0.002] |
| Number of observations | 715990 | | 956322 | |  |  |

**Table A5** Table 5: The impact of VFP on rural unemployment

|  | Dependent variable: Unemployment | | | | | |
| --- | --- | --- | --- | --- | --- | --- |
|  | [1] | [2] | [3] | [4] | [5] | [6] |
|  |  |  |  |  |  |  |
| Post | -0.043 | -0.031 | -0.047 | 0.086 | 0.092 | 0.091 |
|  | (0.001)*** | (0.001)*** | (0.001)*** | (0.000)*** | (0.000)*** | (0.000)*** |
| *Desa* | -0.117 | -0.068 | -0.054 | 0.007 | 0.019 | 0.019 |
|  | (0.001)*** | (0.001)*** | (0.001)*** | (0.000)*** | (0.000)*** | (0.000)*** |
| Post X *Desa* | 0.007 | -0.000 | -0.001 | -0.023 | -0.025 | -0.025 |
|  | (0.001)*** | (0.001) | (0.001) | (0.001)*** | (0.001)*** | (0.001)*** |
|  |  |  |  |  |  |  |
| Number of observations | 2,250,608 | 2,250,608 | 2,250,608 | 2,250,608 | 2,250,608 | 2,250,608 |
| *R^2^* | 0.029 | 0.289 | 0.301 | 0.858 | 0.868 | 0.868 |
|  |  |  |  |  |  |  |
| District Fixed Effect | Y | Y | Y | Y | Y | Y |
| Control variables include: |  |  |  |  |  |  |
| Personal characteristics | N | Y | Y | Y | Y | Y |
| Education | N | N | Y | Y | Y | Y |
| Economic Sectors | N | N | N | Y | Y | Y |
| Head of the households | N | N | N | N | Y | Y |
| Member of household | N | N | N | N | N | Y |

Notes: This table presents the impact of the VFP on village labour market. The outcome variable is indicator variable that is 1 if labor force is not working and 0 otherwise. Control variables included in the model are presented in the Table 1 and 2. All standard errors are clustered at the household level. *,**, and *** represent statistical significance at 10, 5, and 1 percent, respectively.
